# The Importance of Controlled Mismatch of Biomechanical Compliances of Implantable Scaffolds and Native Tissue for Articular Cartilage Regeneration

**DOI:** 10.3389/fbioe.2018.00187

**Published:** 2018-11-30

**Authors:** Michael Gasik, Alexandra Zühlke, Anne-Marie Haaparanta, Virpi Muhonen, Kaisa Laine, Yevgen Bilotsky, Minna Kellomäki, Ilkka Kiviranta

**Affiliations:** ^1^School of Chemical Engineering Aalto University Foundation, Espoo, Finland; ^2^Seqvera Ltd., Helsinki, Finland; ^3^BioMediTech and Faculty of Biomedical Sciences and Engineering Tampere University of Technology, Tampere, Finland; ^4^Department of Orthopaedics and Traumatology, University of Helsinki and Helsinki University Hospital, Helsinki, Finland; ^5^BioMediTech and Faculty of Life Sciences and Medicine University of Tampere, Tampere, Finland

**Keywords:** articular cartilage, scaffold, PLA, collagen, biomechanics, testing, synovial fluid

## Abstract

Scaffolds for articular cartilage repair have to be optimally biodegradable with simultaneous promotion of hyaline cartilage formation under rather complex biomechanical and physiological conditions. It has been generally accepted that scaffold structure and composition would be the best when it mimics the structure of native cartilage. However, a reparative construct mimicking the mature native tissue in a healing tissue site presents a biological mismatch of reparative stimuli. In this work, we studied a new recombinant human type III collagen-polylactide (rhCol-PLA) scaffolds. The rhCol-PLA scaffolds were assessed for their relative performance in simulated synovial fluids of 1 and 4 mg/mL sodium hyaluronate with application of model-free analysis with Biomaterials Enhanced Simulation Test (BEST). Pure PLA scaffold was used as a control. The BEST results were compared to the results of a prior *in vivo* study with rhCol-PLA. Collectively the data indicated that a successful articular cartilage repair require lower stiffness of the scaffold compared to surrounding cartilage yet matching the strain compliance both in static and dynamic conditions. This ensures an optimal combination of load transfer and effective oscillatory nutrients supply to the cells. The results encourage further development of intelligent scaffold structures for optimal articular cartilage repair rather than simply trying to imitate the respective original tissue.

## Introduction

The need to develop tissue substitutes and regeneration platforms is one of the most demanding and challenging applications in modern tissue engineering (Hubbell, 1995; Burdick and Mauck, 2011). Three-dimensional biomaterial structures (scaffolds) are highly desirable matching the biomechanical properties of the tissue (Gomes and Reis, 2004) and closely mimicking *in vivo* behavior [facilitating cell adhesion, growth, and tissue formation (Volfson et al., 2008)]. Such biomaterials assist the body to rebuild the damaged tissue and eventually they minimize associated pain and healing time (Wong and Bronzino, 2007; Chung and Burdick, 2008). The combined static and dynamic biomechanical properties of these scaffolds are crucial for the final success of the treatment. Any progress in the development of scaffolds should ensure a high correlation between *in vitro* conditions and expected *in vivo* tissue regeneration (Frost, 2004; Wilson et al., 2006; Mollon et al., 2013). The non-toxic biodegradation of the scaffold should gradually transfer the stress to the new growing tissue over an appropriate time period. As pointed out recently (Panadero et al., 2016), the synergetic effect of correct mechanical stimulation is greatly dependent on the scaffolding material, its environment and the cell presence. This shows the needs for consistent simultaneous analysis to compare different biomaterials and to get conclusions about these features.

One of the most challenging applications of biomedical scaffolds is the articular cartilage (AC) repair. The damage and degradation of AC are not only progressing with age, obesity, or systemic diseases, but also in the young and active population due to physical causes, such as injury. If untreated, these defects may progress toward osteoarthritis (OA), affecting over 150 million people worldwide, mainly by degeneration of the hyaline cartilage in synovial joint lacking the ability of self-regeneration (Armstrong and Mow, 1982). Natural wound healing, in full-thickness defects of cartilage, often leads to the formation of fibrocartilage (Armstrong and Mow, 1982; Wilson et al., 2006; Mollon et al., 2013; Panadero et al., 2016), which is functionally and biomechanically inferior to the original hyaline cartilage making the tissue more prone to further deterioration and osteoarthritic changes of the joint. Initiated vicious cycle (Benders et al., 2012) ultimately will call for a total or partial joint replacement. Therefore, chondro-conductive and -inductive biomaterials are highly desirable to treat cartilage lesions at early stages before manifestation of OA.

Clinically used biomaterials include various naturally derived and synthetic materials (von Recum, 1998; Agrawal and Parr JE, 2000). The advantage of natural materials is their intrinsic bioactivity for the purpose, although application of animal-derived materials (xenografts) contains certain risks, such as contamination and undesired immune response. This could be avoided by using bioabsorbable synthetic materials not causing foreign body or hypersensitivity reactions themselves. Synthetic materials can be made biologically more advantageous and biocompatible. On the other hand, compared to the naturally derived materials, synthetic polymers are usually lacking the desired intrinsic biological cues that promote cell adhesion, proliferation and tissue recovery. However, any biomaterial is always challenging to evaluate and optimize for clinical use and for the purpose aiming on “precise medicine” solutions. It is now widely anticipated that the present level to evaluate the mechanical function of biomaterial and tissue engineering constructs is highly insufficient. For example, of 205 analyzed articles on cartilage tissue engineering, mentioning of applied mechanical stimulation, only 29% shows some quantified material properties (Lujan et al., 2011). Correct and detailed biomaterial testing is rather time-consuming and expertise to properly quantify non-elastic material behavior of tissue is also scarce in many dedicated biology labs (Lujan et al., 2011).

Synthetic materials with fibrous origin are often used for AC repair applications. These scaffolds have 75–85% porosity and they are exposed to synovial fluid with sodium hyaluronate (NaHA). Animal studies are needed to ensure the biological functionality of the scaffolds before clinical use. However, the relationship between the natural tissue and the scaffold is challenging to measure. The regulations and the worldwide trends impose more pressure to move from animal models into *in vitro* evaluation (Directive 2010/63/EC for Alternative Methods, 2015). Therefore, in order to develop and optimize biomaterials, one must establish protocols for reliable comparison of different materials before *in vivo* tests can be ethically justified and their results truly extrapolated toward safe and effective human use.

The structure, functions and biomechanical behavior of AC are very complex, highly anisotropic and time- and loading history-dependent (Wilson et al., 2005). The articular cartilage consists of a relatively small number of chondrocytes surrounded by a multi-component matrix, which can be imaged as a composite with 70–85% water and remaining proteoglycans (proteins with glycosaminoglycans attached as a bottlebrush-like structure) and collagen (Hayes, 1972). Proteoglycans and water concentration vary through the depth of the cartilage tissue. Proteoglycans can bind or aggregate to a backbone of sodium hyaluronate (NaHA) of molecular weight of 2–4 MDa to form a macromolecule weighting up to 200 MDa (Kobayashi et al., 1994).

The biomechanics of AC and synovial fluid is also complex and essentially non-linear (Hayes and Mockros, 1971; Hayes and Bodine, 1978). Not some many studies have coherently and systematically analyzed AC properties (Ahsan and Sah, 1999; Korhonen et al., 2006) due to variability of the samples, local inhomogeneity and applied biomechanical methods. Complex loading schemes are associated with significant variations of interstitial fluid pressure and fluid flow, complicating the results interpretation (Ahsan and Sah, 1999). The collagen-rich matrix behavior is highly non-linear and requires rather sophisticated models to be described as a composite material, whether with theories (Mäkelä and Korhonen, 2016). Synovial fluid is well-known to have non-Newtonian viscosity vs. its composition, shear rate, mode of loading and the presence of other factors (King, 1966). Most of the biomechanical properties of AC tissue reported experimentally are obtained with either confined compression (Mow et al., 1980) or indentation (Kempson et al., 1971). These measurements data are commonly approximated with biphasic (Mak et al., 1987) or triphasic (Lai et al., 1991) theories, or even more simplified viscoelastic models. However, due to peculiarities of the AC tissue properties (Lai et al., 1981), it is difficult to compare results published with different studies, using various specimen types, methods and testing devices. It was also reported (Hosseini et al., 2014) that fluid flow and flow-dependent phenomena may dominate the AC behavior in different testing regimes and thus it is impossible to determine in general required recovery time. Aggregate modulus in range of 50–120 kPa was reported for human, bovine and canine tissues by different sources (Hayes and Mockros, 1971; Kempson et al., 1971; Hayes, 1972; Mow et al., 1980; Lai et al., 1981; Armstrong and Mow, 1982; Wilson et al., 2006), but often full test data were not available to compare these data [indentation usually produces much larger values (Ahsan and Sah, 1999; Korhonen et al., 2006)]. Formal models of AC are missing essential features which limit their practical application only to specimens analyzed in that studies. Therefore, it is a great oversimplification to characterize AC or scaffolds for AC repair by set of one or two numbers without exact data on the test method and data analysis.

In this study we used highly porous PLA mesh manufactured from fine PLA fibers. Even though PLA itself is a stiff material, this studied PLA mesh was optimized to have a relatively soft nature to suit better as cartilage repair matrix. The hypothesis was that a scaffold which is less stiff than surrounding tissue and which is acting in compression under the requirement of strain compliance will have less stress and therefore fluid pressure which would cause fluid to flow into the scaffold to bring more nutrients to chondrocytes. The collagen component was added to the PLA mesh to increase the hydrophilic nature of the scaffolds and to promote cell proliferation (Muhonen et al., 2016; Gasik et al., 2017). Here we report results of this new xeno-free, recombinant human collagen-laden (rhCol) polylactide (PLA) mesh scaffolds (rhCol-PLA) developed for repair of early cartilage lesions to avoid osteoarthritic changes, which have been designed, produced, and biomechanically optimized *in vitro* and *in vivo* validated in equine (unpublished data) and porcine models (Muhonen et al., 2016; Gasik et al., 2017). The rhCol-PLA scaffolds were assessed for their relative performance in simulated synovial fluids for mimicking both human and veterinary conditions with application of model-free analysis with Biomaterials Enhanced Simulation Test (BEST). The results of the scaffold materials selection were also correlated with *in vivo* tests, carried out in a separate study (Haaparanta et al., 2014; Muhonen et al., 2016), where this material combination was found to work better than the previously studied plain PLA scaffolds with stiffer structure (Pulliainen et al., 2007).

## Materials and Methods

### Materials Analyzed

The scaffolds tested were made of synthetic polymer fibers. The polylactide scaffold (PLA) was processed of medical grade poly-(L/D)-lactide PLA96/4 (Corbion Purac, Gorinchem, NED), manufactured to melt spun fibers and afterwards carded and needle punched into meshes (porosity ~90–93%) in Tampere University of Technology (Tampere, Finland). The used PLA was a highly purified, medical grade polymer (Länsman et al., 2006) with a residual monomer content of <0.5%. The PLA meshes were washed with ethanol, dried, packed and sterilized by gamma irradiation 25 kGy. A part of PLA scaffolds was aseptically doped (Haaparanta et al., 2014) with a recombinant human collagen III (FibroGen, Inc., San Francisco, USA) solution and the structure was freeze-dried (marked as rhCol-PLA). The rhCol-PLA scaffolds were further crosslinked with 14 mM 1-ethyl-3-(3-dimethylaminopropyl)-carbodiimide hydrochloride (EDC) + 6 mM N-hydroxysuccinimide (NHS) (Sigma-Aldrich, Helsinki, Finland) in 95% ethanol, washed and subsequently freeze-dried again. The ratio between the PLA and collagen components in the rhCol-PLA scaffolds was 86/14 vol. % of PLA and collagen, respectively.

All the specimens of PLA and rhCol-PLA scaffolds were cut into rectangular pieces ~5 × 5 mm (±1 mm) in size with the thickness of the original materials as supplied. The exact size of the specimens was measured with a non-contact method using a laser micrometer (MetraLight, CA, USA) with ±1 μm resolution and the samples were weighted with a balance before and after the test. The measuring and weighing process was repeated three times; on the dry sample, on the immersed sample and on the sample after the measurement. The samples were immersed in distilled water to ensure that the sample was completely wet before inserting to the sample holder as possible trapped air bubbles may alter the test results improperly.

The media-simulated synovial fluid (SSF)—for the tests was prepared as two solutions with different concentrations of sodium hyaluronate (NaHA). Sodium hyaluronate of molecular weight 1.68 MDa (Nutrihyl®, Contipro Biotech, Czech Republic) was dissolved in 200 mL of cold distilled water to mimic “normal” (4 mg/mL) and “osteoarthritic” (1 mg/mL) solutions (Fam et al., 2007).

### Experimental Methods

The viscosity of the SSF solutions was determined using SV-10 vibro-viscosimeter (A&D Co. Ltd., JAP) consisted of two vibrating gold plates immersed in the solution. About 45 mL of the SSF solution was poured in a cuvette and heated to 40°C. The cuvette was then placed at the viscometer and the viscosity with temperature was measured simultaneously upon free cooling. Viscosity curves were well-fitted for every composition with the Arrhenius equation.

The biomechanical analysis was carried out using two dynamic mechanical analysis (DMA) 242C and 242E machines (Netzsch Gerätebau GmbH, Germany) with a specially developed biomaterials enhanced simulation test (BEST; Seqvera Ltd., Finland) protocol (Gasik, 2014, 2017a,b), adjusted for simulated cartilage conditions (Hayes and Bodine, 1978; Mow et al., 1980; Lai et al., 1981). The compressive mode sample holder and the specimen were fully immersed in the thermally controlled bath with media (~30 mL). This resembles the gradients of deformation, pore pressure and fluid flow similar to tibial cartilage conditions as has been shown with other experiments and computer simulations (Korhonen et al, 2002; Milan et al., 2010). Three different protocols were applied for immersion tests: creep (*n* = 38 for PLA and *n* = 40 for rhCol-PLA), frequency scans (0.01–20 Hz) as *n* = 38 for PLA and *n* = 28 for rhCol-PLA, and strain sweeps up to 25–50 μm at 1 Hz) as *n* = 20 for PLA and *n* = 30 for rhCol-PLA.

A preconditioning step was applied (Pioletti and Rakotomanana, 2000) to all specimens by an axial confinement by ~5 μm of initial deformation (offset), following the 15 min equilibration under a small force of 0.05 N to stabilize the dimensions and temperature. This was found to suppress initial swelling (where present; as explained below) thus all the creep deformation and compliance are originated from zero. At these conditions it was observed that deformation of the porous, fully saturated fibrous structures proceeded without excessive deformation of the fibers themselves and without substantial decrease in porosity. Whereas, *in vivo* pressures expressed on healthy articular cartilage may reach 1–10 MPa at peak, here the fluid flow is unconfined but the scaffold as AC tissue undergoes deformation similar to one in clinical conditions (Lai et al., 1981, 1991; Milan et al., 2010).

### Data Analysis

Experimental data has been converted into biomechanical values and analyzed with an application of idempotent type analysis without use of a material model (Gunawardena, 1996; Pioletti and Rakotomanana, 2000; Litvinov et al., 2001). This gives an advantage over commonly reported moduli functional dependence as it allows extraction of the time-invariant data suitable for future prediction of the material behavior. The data quality reported for the same material might be also confusing, as no exact information is given for conditioning changes, and usually no solid proof shown, e.g., about suitability of the small strain theory or material linearity (Norris, 2008; Lujan et al., 2011). Such conditions are often assumed by default, despite it is of common knowledge that “elastic modulus” cannot be uniquely defined for material which does not follow linear elasticity model.

Idempotent processing, common in computer technology (Gunawardena, 1996) preserves the data structure and original variables without demand of explicit knowledge of their functional dependencies. It obeys causality principle (response always comes after the stimulus applied) and respects the boundaries of thermodynamics (no violation of conservation laws). One essential advantage in testing of biomaterials with this method is in taking into account non-local effects—on the contrary to conventional mathematical analysis, where the derivative of a function is always local. Hence, the predictors can be used in *in silico* simulations to calculate, for example, mechano-regulative index without necessity of explicit local fluid flow determination (Maslov, 1970; Gasik, 2017b).

Stress was calculated as Piola–Kirchhoff stress, from the ratio of acting force to the original surface area A_0_ of the specimen, σ = *F/A*_0_. The strain was calculated as Lagrange (true logarithmic) strain, related to the initial thickness *H*_0_ at the beginning of the creep: ε*(t)* = *ln(1* + Δ*L(t)/H*_0_*)*, where Δ*L(t)* is the observed change in the specimen thickness with experiment time *t*. Whereas, other strain definitions can be also used, this one has a rigorous thermodynamic rationale (Xiao, 1995; Lubarda and Chen, 2008). The ratio of the strain ε*(t)*, to constant stress, σ_0_, is the creep compliance *C(t)* = ε*(t)/*σ_0_, which is the main readout from the creep experiments. For dynamic loading, the strain amplitude is from a harmonic signal waveform extreme, taking into account load history:

(1) $$\begin{array}{r} \varepsilon_{\omega }\left(t\right)=\frac{1}{2}\ln\;\left(\frac{H_{0}+\Delta L\left(t\right)+a_{0}}{H_{0}+\Delta L\left(t\right)-a_{0}}\right) \end{array}$$

where *a*_0_ is the applied deformation amplitude at some instant frequency ω. In this format, the irreversible creep or similar deformation is taken into account for long experiment times (*t*). All experiments were preformed in triplicate and for every frequency or strain test 10 cycles were used within every run. Hence for time dependencies one should consider both long time (real time of the test—minutes and hours) and short time (time span within one or few dynamic cycles—seconds).

Test readouts from DMA experiments have been processed with model-free idempotent methods (Gasik and Bilotsky, 2018). In general, there is no explicit mathematical formula written as the calculation is iteratively progressing for every data point collected. This allows inclusion of specimen history without a need of assumption of time kernels (hereditary integrals). In this work the approach was used to find for instance aggregate modulus, material memory, static and dynamic permeability.

## Results

### Materials Preparation and Preliminary Analysis

The viscosity of SSF compositions was approximated as function of temperature with Arrhenius equation, leading to 8.14·10^−4^·exp(3022.2/T) and 1.746·exp(1143/T) in mPa·s, for 1 and 4 mg/mL NaHA, respectively (correlation *r*^2^ = 0.9869–0.9953). At 25°C this gives 20.5 and 80.9 mPa·s values for these SSF (for comparison, water viscosity is 0.89 mPa·s). The exact values are not explicitly required because the DMA device and sample holder dynamics have been recalibrated for every type of SSF, and thus media viscosity changes have been automatically included in the test data.

**Figure 1:**
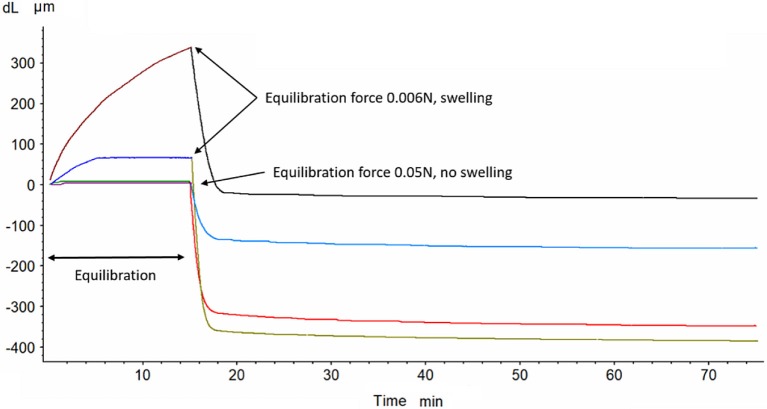
The effect of conditioning force (0.006 and 0.05 N) on swelling and compaction (at 0.2 N) of PLA scaffold (two experimental curves for either forces combination).

At the beginning of the tests it was discovered that PLA materials exhibit very high swelling ratios even if they were completely soaked in liquid before the tests. This is usually faced in hydrophobic materials at free swelling due to repulsive forces and pressure variations (Bennethum and Weinstein, 2004). It was formulated (Bennethum and Cushman, 1996) that the macroscopic solid stress tensor (related to visible bulk swelling) is combined of a thermodynamic solid pressure, a solid stress tensor, a stress due to the interaction of the solid and liquid phases, and a stress due to the interaction of the interface with the solid phase as well as kinetic component of constituents. By changing the conditioning pressure, it was found that the force of ~50 mN (equivalent to applied ~1 kPa stress) is required to suppress the swelling but not to cause pre-compression of the sample, Figure 1. This conditioning pressure was used in all these experiments to get consistent results.

### Pseudo-Static Experiments

**Figure 2:**
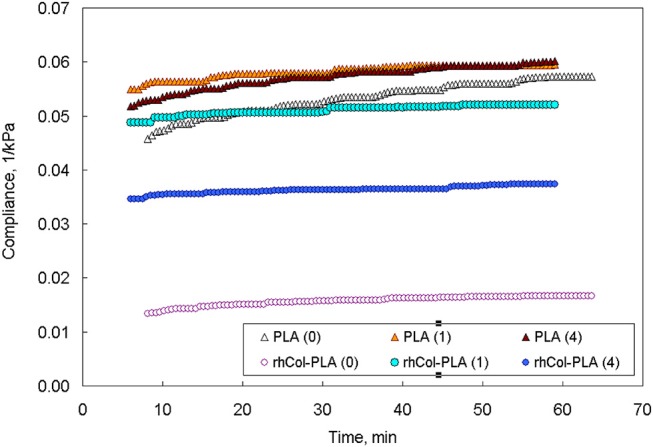
Creep compliance at 0.2 N for P LA and rhCol-PLA in different media (numbers in parentheses show NaHA concentration in SSF, mg/mL).

The results of one set of creep measurements at 0.2 N (~4 kPa applied stress) are shown in Figure 2. The level of applied stress of 4 kPa (~30 mmHg) was considered to be a limit which does not cause cartilage-adjacent soft tissues necrosis (Goode and Shinn, 1977). The nature of a creep test is pseudo-static (change of strain in time at constant applied stress) and it is often used to evaluate viscoelastic nature of materials and to approximate it with some models (Bilotsky and Gasik, 2015). Here one may see that addition of NaHA to media does not affect compliance of PLA much, but has a great effect on rhCol-PLA. It is notable that compliance of rhCol-PLA in 1 mg/mL SSF is the highest, and in 4 mg/mL is average between 1 mg/mL and water (0 mg/mL NaHA). The higher is the compliance, the more easily the material deforms under constant load. Hence an observation can be drawn that addition of cross-linked collagen to PLA makes it “stiffer” when tested in water but makes little difference when testing in 1 mg/mL SSF.

**Figure 3:**
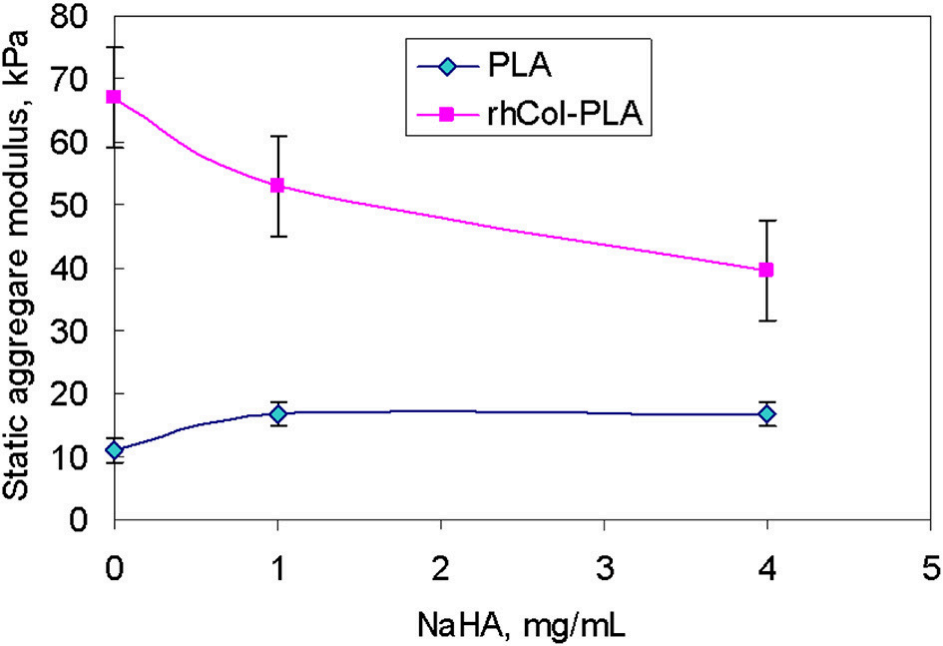
Static aggregate modulus (kPa) of the scaffolds vs. NaHA concentration in SSF. Bars here and further indicate standard error unless stated otherwise.

**Figure 4:**
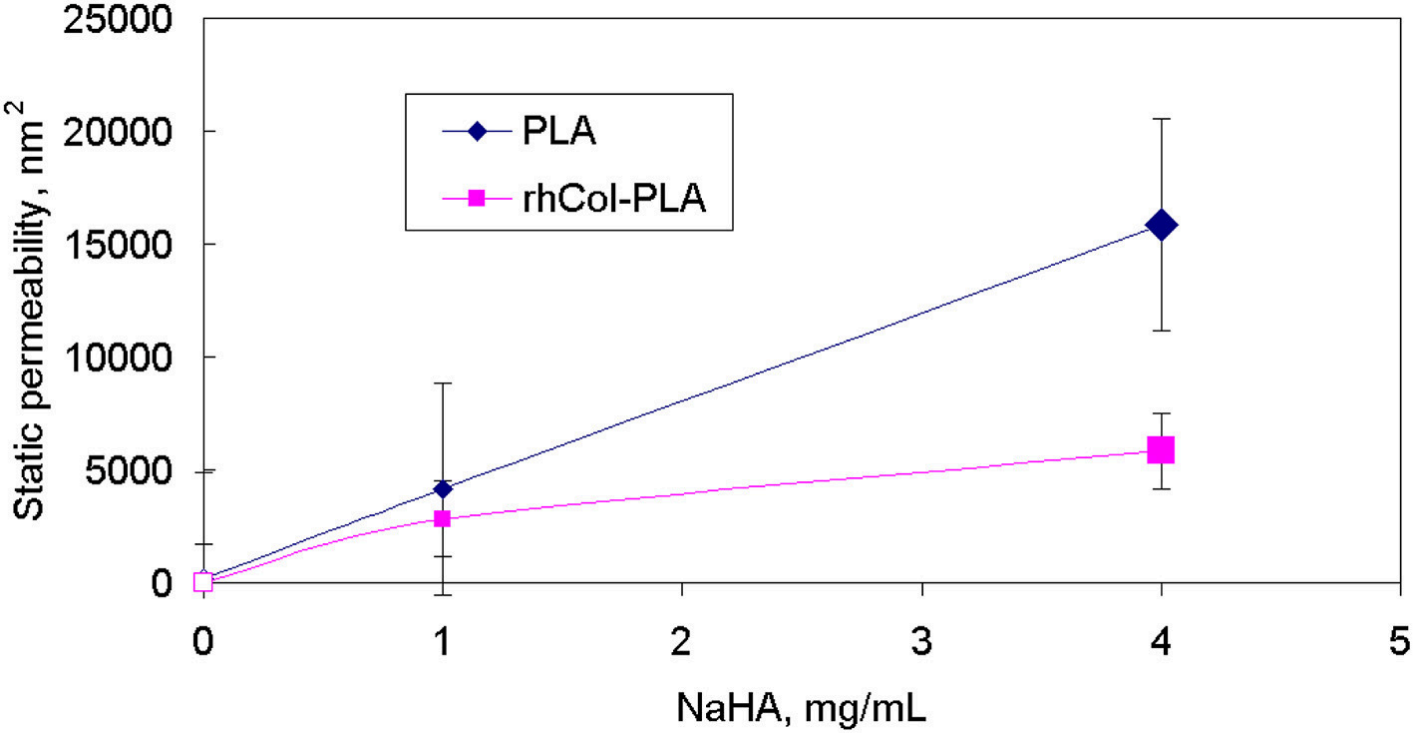
Static (creep) permeability of scaffolds vs. NaHA concentration in SSF, indicating that static permeability increases at higher NaHA concentration.

The comparison of the data from pseudo-static (creep) analysis (Figure 3) shows that stiffness of the rhCol-PLA scaffolds is increased by several times when compared to PLA, and this effect is independent on the type of SSF used. Also rhCol-PLA material in static conditions has lower permeability (Figure 4), which in combination supports a vision that synovial fluid will likely be kept in rhCol-PLA better than in PLA—at the same loading, within the same time span PLA will lose fluid to a greater extent.

### Frequency Experiments

Behavior of materials under constant deformation but varied frequency is different from pseudo-static one. These differences in the case of fibrous porous materials are due: (1) oscillating mobility of fluid within a fibrous structure, (2) inertia effects associated with hysteresis between incoming and outgoing fluid flow, and (3) non-linearity in fluid viscous properties and possible non-linearity in coupled deformation of the fibrous skeleton of the scaffold.

**Figure 5:**
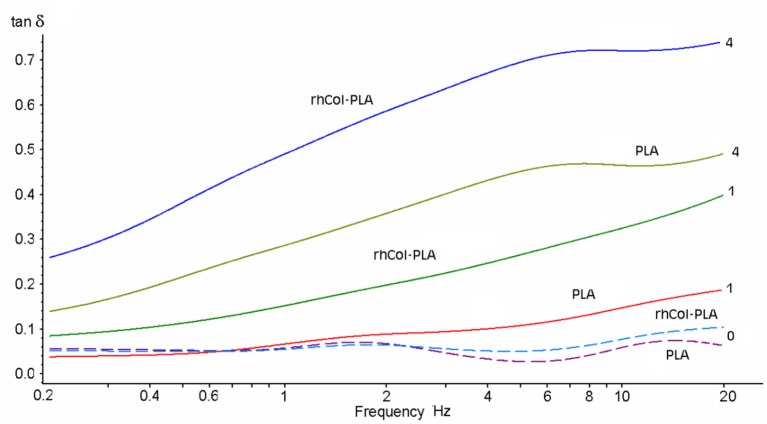
Loss tangent of the scaffolds in different SSF (NaHA concentration as numbers, mg/mL) vs. frequency at 25 μm deformation amplitude.

**Figure 6:**
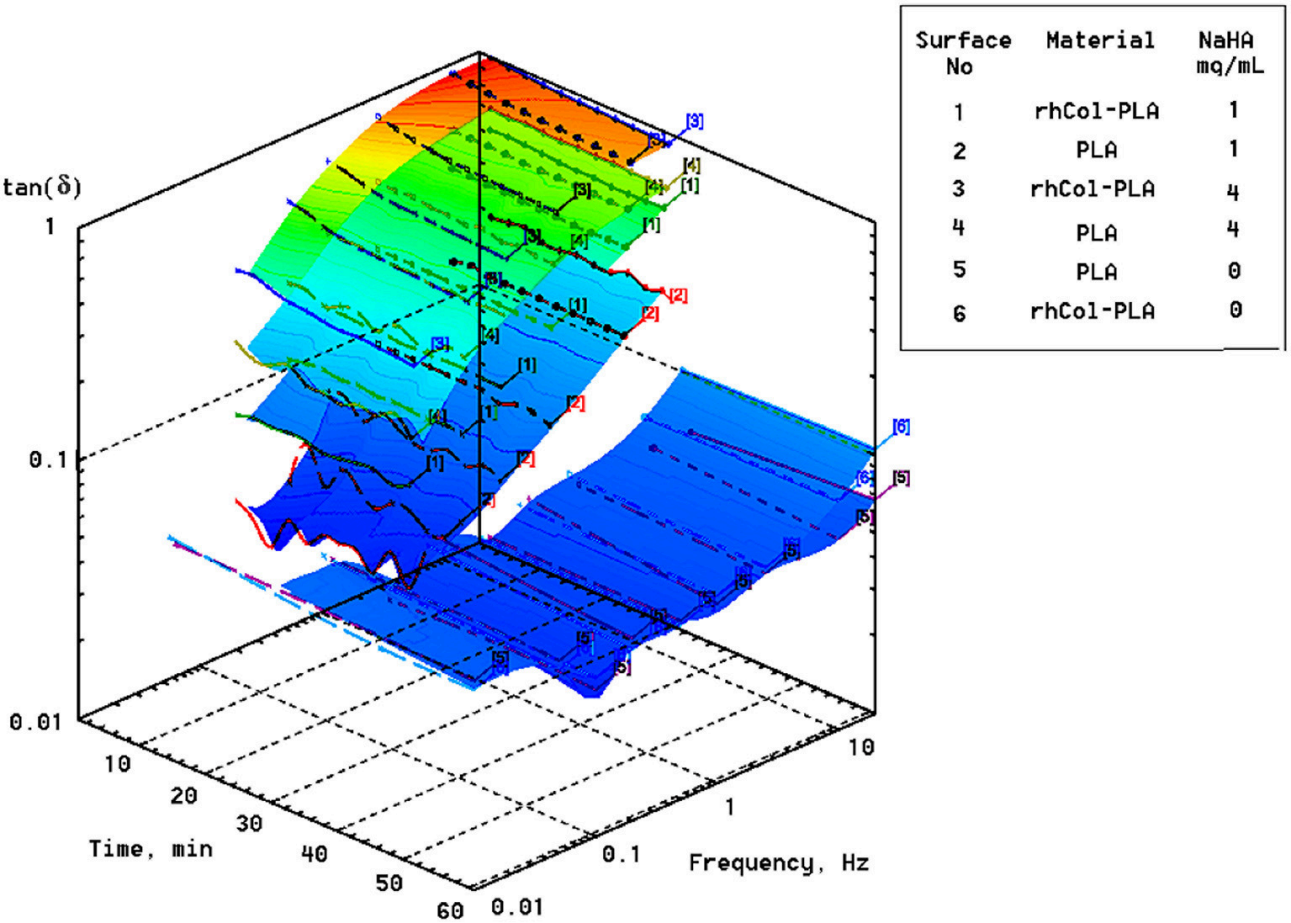
Loss factor [tan(δ)] of scaffolds in different SSF vs. applied frequency at 25 μm displacement amplitude. Note plots for water (0 mg/mL NaHA; surfaces No. 5 and 6) are shifted ahead of time scale to improve visual readability of the plots.

One of the experimental criteria to observe the differences is the loss tangent [tan(δ)], which is defined as the ratio of imaginary to real part of elastic moduli or stiffness. Higher loss tangent of rhCol-PLA vs. PLA (Figure 5) was observed for all frequencies. Figure 6 shows a 3D plot of these dependencies of tan(δ) vs. applied frequency at 25 μm deformation amplitude along with experiment time (note the tangent is practically constant with the time and depends essentially on frequency only).

It is seen that addition of NaHA to the media and respective increase in viscosity also leads to increase in the loss tangent (the more, the higher is the NaHA concentration). For rhCol-PLA loss tangent is roughly 2–3 times higher than for PLA in all SSF (Figure 6). This indicates more active interaction of fluid flow with rhCol-PLA than PLA and is likely associated with a finest collagen fibrils network between the PLA-based fibers in rhCol-PLA (Länsman et al., 2006; Muhonen et al., 2016; Gasik et al., 2017). Notable is that this interaction is only due to presence of NaHA, as such differences are not seen when only water is used (Figure 6).

### Strain Sweep Experiments

One of the more physiologically relevant dynamic conditions is application of variable deformation under constant frequency. This can be depicted as change of the gait loads keeping normal walking conditions (~1 Hz) (Hayes and Mockros, 1971; Hayes, 1972; Mow et al., 1980; Armstrong and Mow, 1982). Thus, the last test sequence was applied to simulate changes in properties of scaffolds up to 50 μm of dynamic deformation with repeating of the load cycles. At constant frequency, loss tangent is not significantly deformation- or stress-dependent so here major performance comes from dynamic stiffness and fluid exchange.

**Figure 7:**
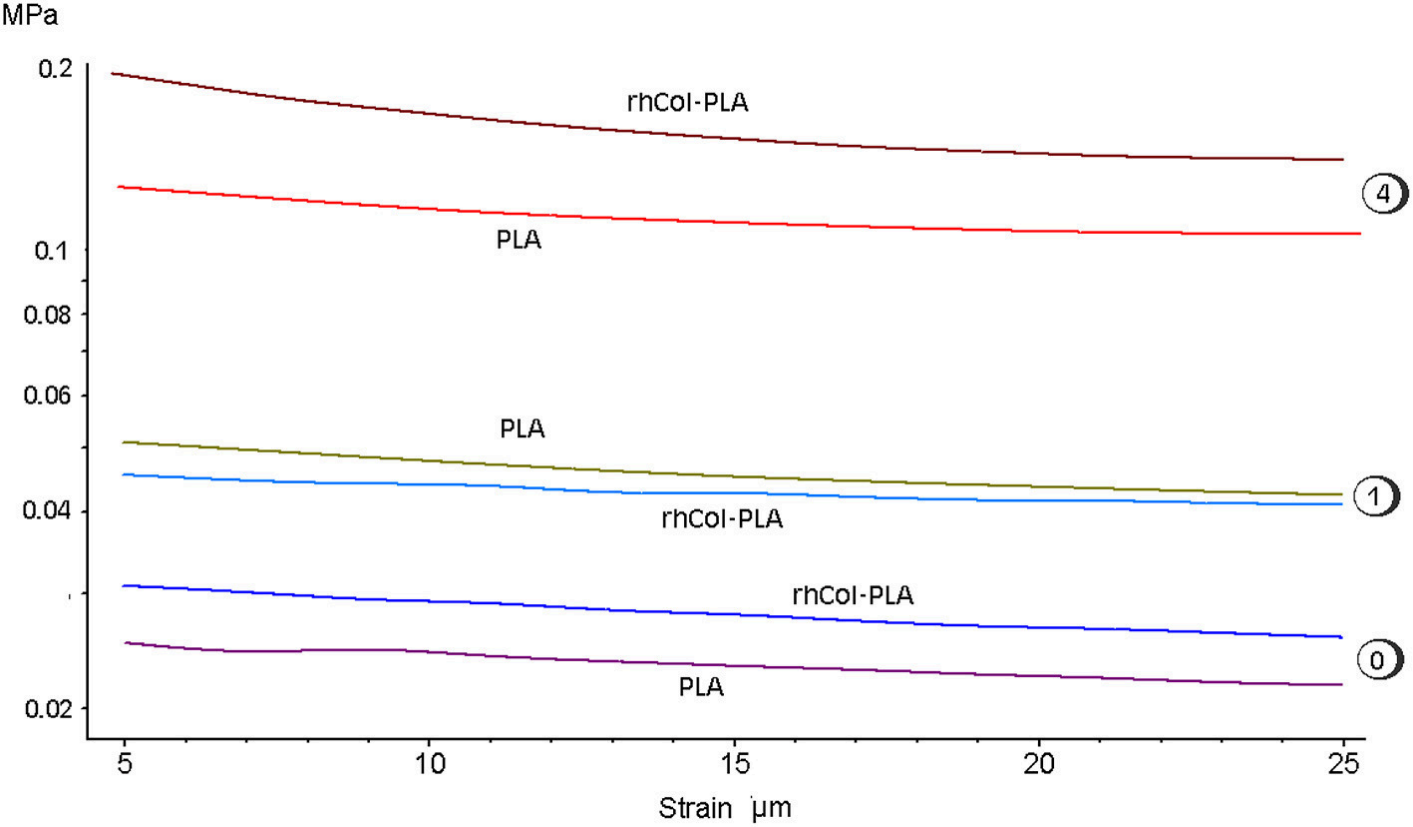
Average dynamic modulus of scaffolds at 1 Hz vs. applied deformation ad different SSF (numbers indicating NaHA concentration, mg/mL). Note log scale for modulus.

**Figure 8:**
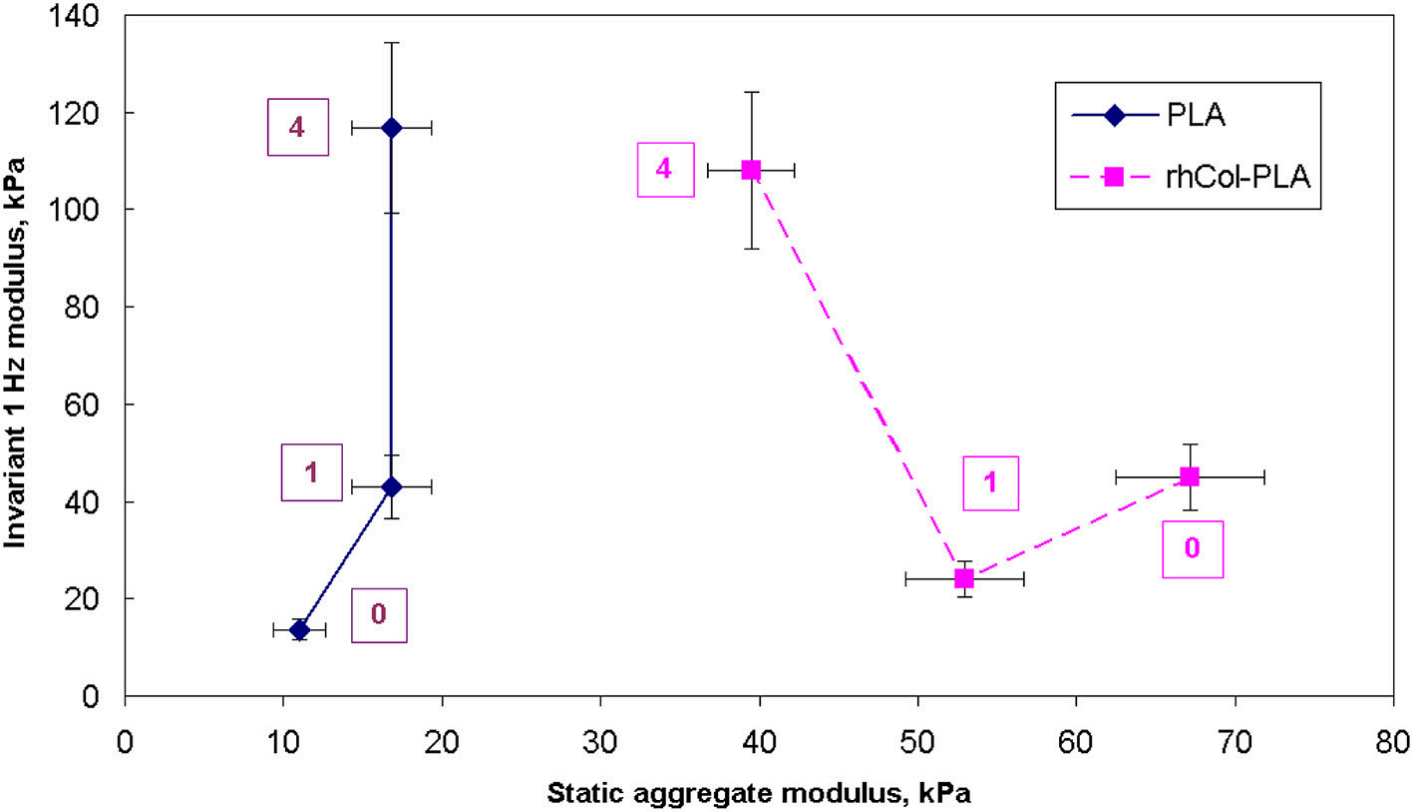
Comparison of static aggregate modulus (Figure 3) with invariant dynamic modulus at 1 Hz. Numbers indicate concentration of NaHA in SSF, mg/mL.

**Figure 9:**
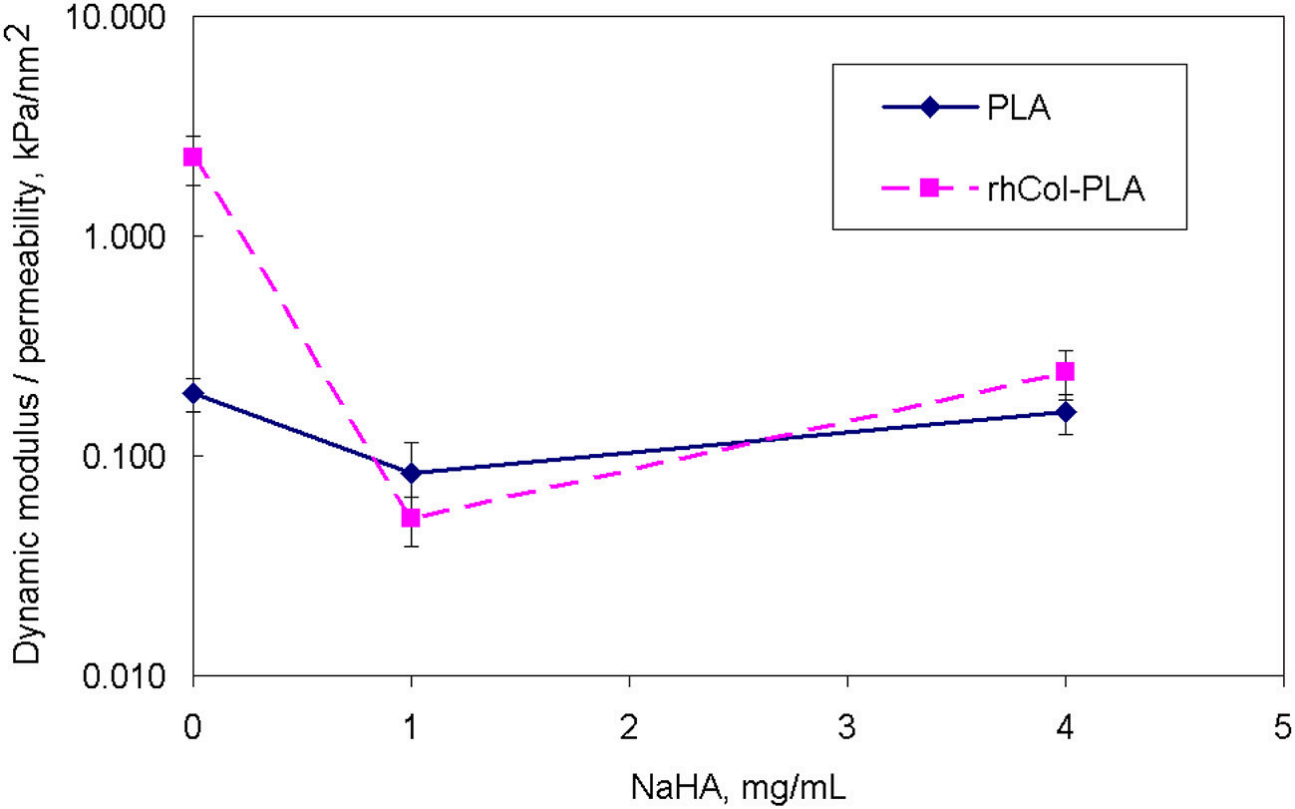
Comparison of ratio of dynamic invariant modulus to dynamic permeability vs. NaHA concentration (note log scale).

The absolute value of average dynamic elastic modulus at 1 Hz is shown in Figure 7 as directly obtained from the DMA signal. It is seen that this modulus slightly decreases with deformation. However, with increased number of loading cycles and true strain variations due to changes in geometry, true (corrected) elastic modulus slightly increases. Also, higher NaHA concentration shows higher stiffness of all materials but it is noteworthy this stiffness incorporated fluid movement under dynamic load and therefore unavoidably includes some viscous and inertia contribution, as shown about for loss tangent (Figures 5, 6). Analyzed invariant modulus at 1 Hz is shown in Figure 8 vs. respective static aggregate modulus (Figure 3) for respective NaHA concentration. The ratio of dynamic modulus to dynamic permeability is shown in Figure 9 for 1 Hz condition. Here rhCol-PLA is at least similar or better (at 1 mg/mL NaHA) than PLA scaffolds.

## Discussion

The two studied scaffolds contained the same kind of PLA mesh structures. The PLA scaffolds were studied as such and in the rhCol-PLA scaffolds the collagen component was added into the structure to give the highly porous PLA scaffold increased hydrophilic nature and to promote cell proliferation. From the post-processing of the experimental data, many additional values have been obtained without assumption of a material model (a proprietary patent-pending method). Here data for aggregate modulus (in static and dynamics) and permeability are shown as an example.

**Figure 10:**
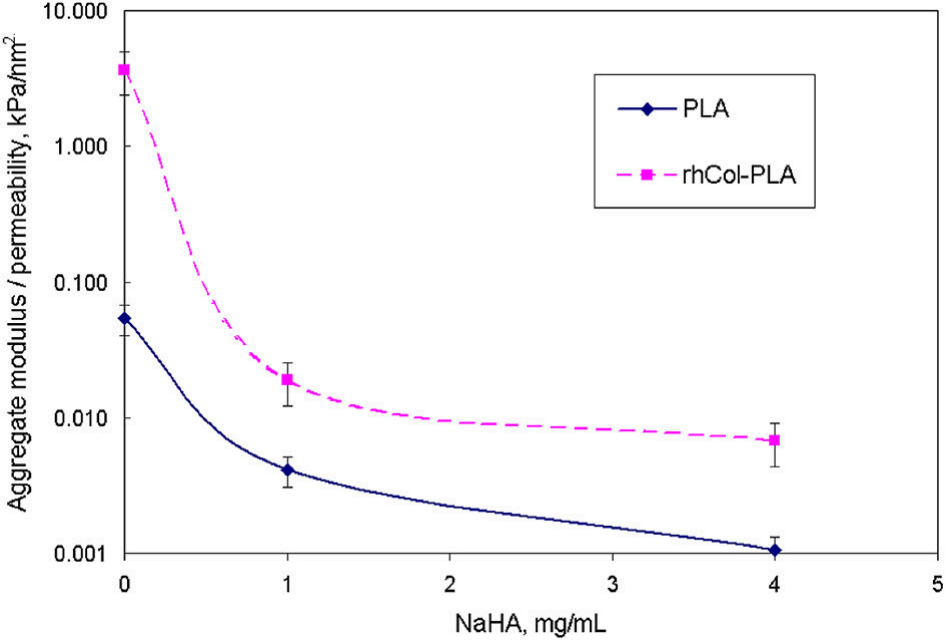
Comparison of ratio of static aggregate modulus (Figure 3) to permeability (Figure 4) vs. NaHA concentration (note log scale).

The relevancy of static conditions results for clinical conditions is that rhCol-PLA scaffolds are better supportive for weight-bearing and undergo smaller deformation than pure PLA. In combination of lower permeability this suggests synovial fluid to stay likely rhCol-PLA more than in PLA, whereas the latter will lose more fluid at the same loading. As a simple decision-aiding criterion, one might consider the ratio of aggregate modulus to permeability: the higher it is, the better the scaffold withstands static loads. Figure 10 shows that in this respect rhCol-PLA material is by 1–2 orders of magnitude superior to PLA at all tested SSF compositions.

At dynamic conditions, such as walking, the situation reverses: under dynamic loading one has to aim on more active fluid exchange to provide biomechanical stimulus to chondrocytes, to ensure fluid and nutrients supply and removal of metabolic products, promoting tissue regeneration (Gerisch and Chaplain, 2006; Sittichokechaiwut et al., 2010). Cartilage tissue is avascular and its extracellular matrix creates further barriers for nutrient/waste exchange by diffusion. Thus, lower dynamic modulus (Figure 8) and better fluid diffusivities are desired. These features have to be, however, compatible to the above requirements for static conditions as cartilage must work well in both these extremes.

The viscosities of SSF are substantially higher than water: about 20 and 100 times for 1 and 4 mg/mL, respectively. This means that even small changes in permeability, i.e., a feature of the material structure, will affect changes in permittivity, a feature of a specific fluid flow thought the material structure. High loss tangent (Figures 5, 6) means more dissipation of applied mechanical energy (inelastic losses) which is important to keep high damping properties of articular cartilage (Mow et al., 1980; Lai et al., 1981; Armstrong and Mow, 1982). Therefore, rhCol-PLA is superior to PLA also in this property, whether for “arthritic” (1 mg/mL NaHA) or “normal” (4 mg/mL NaHA) synovial fluids.

A proof-of-concept animal study was performed in domestic pigs (*Sus scrofa domestica*, 4-months-old, *n* = 20) in a separate study, reported elsewhere (Muhonen et al., 2016). Briefly, the animals were randomized into three groups: (1) rhCol-PLA scaffold treatment, (2) commercial scaffold treatment and (3) spontaneous repair. A circular full-thickness chondral lesion with a diameter of 8 mm was created in the right medial femoral condyle. The removed cartilage tissue was collected and further processed for chondrocyte isolation and subsequent proliferation. After 3 weeks, the lesion was approached again, cleaned and repaired with one of the constructs, i.e., rhCol-PLA or commercial scaffold with chondrocytes, or left untreated. Only one lesion per animal was performed and the animals were allowed free weight-bearing and unrestricted movement after the operations. The repair tissue was evaluated after 4 months. Hyaline cartilage was reported to be formed most frequently in the rhCol-PLA treatment group. Here the analysis of the cartilage repair scores (Haaparanta et al., 2014; Muhonen et al., 2016) was additionally performed using BUGS—Bayesian inference Using Gibbs Sampling (US FDA, 2010), a form of a Markov Chain Monte Carlo sampling. The results of using normal or Poisson distributions of the total normalized ICRS (International Cartilage Repair Society) scores show rhCol-PLA having statistically significant higher average score (0.515) vs. 0.38 for commercial scaffold and 0.288 for spontaneous healing control group (Muhonen et al., 2016).

**Figure 11:**
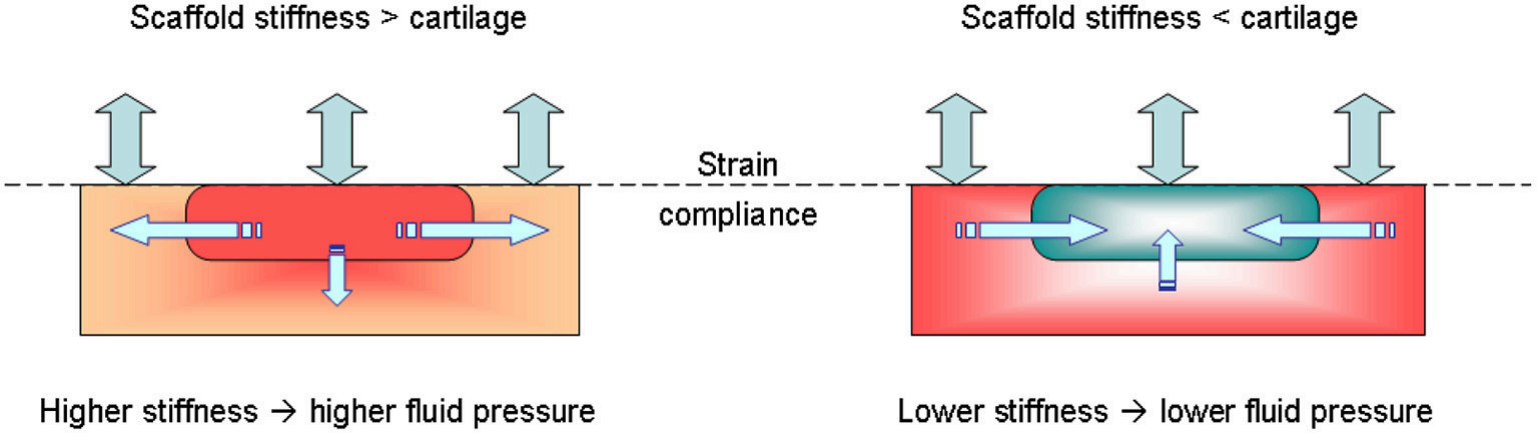
Possible mechanism of beneficial compliance mismatch of scaffolds vs. native tissue when submitted to a mechanical load: when a scaffold with fluid is too stiff, higher generated fluid pressure leads to fluid flow out of scaffold (**left**), whereas for the same deformation conditions of lower stiffness system **(right**) fluid is driven into the scaffold.

A schematic of the advantage of lower dynamic stiffness scaffold for AC repair is depicted in Figure 11. There a scaffold implanted into a cartilage defect should exhibit the same strain compliance (no tears, twisting or buckling). For the same strain, material with a higher apparent stiffness will generate more internal stresses and hence higher fluid pressure (Hayes, 1972; Mow et al., 1980; Lai et al., 1991). This will lead to preferential fluid movement out of cartilage which will not allow cells and tissue regeneration (“dry-out”). For an opposite, lower apparent stiffness (material system with fluid) will have lower fluid pressure which will promote external fluid supply into the scaffold (Bilotsky and Gasik, 2015; Gasik, 2017b; Gasik et al., 2017). The exact values depend on the tissue and scaffold systems differences, fluid properties and loading conditions. Here it is important to note that static and dynamic properties need to be evaluated coherently in a single test to ensure that fluid flow and mechanical compliances are measured linked under proper boundary conditions, and not evaluated separately.

## Conclusions

The results of biomechanical comparison of rhCol-PLA and PLA scaffolds for articular cartilage repair show how both collagen addition and media composition (NaHA concentration) change elastic, viscoelastic and inelastic properties of the scaffolds. Using model-free idempotent analysis methods, it was for the first time possible to extract data on scaffold permeability and invariant moduli in a coherent way, without use of separate experiments. These values make easier design, *in silico* simulation and optimization of such materials.

It might be expressed that for a successful tissue regeneration with scaffolds the properties of the scaffold should not be too close to expected tissue properties, as in this case there will be less stimuli acting on the scaffold for repair. Furthermore, one should consider static and dynamic behavior of the material in the physiologically relevant conditions—the material matching both simultaneously will be the best option to implant.

The studies performed for articular cartilage repair materials could be extended to other cartilage tissues repair implants, as most of these tissues are also subjected to different static and dynamic loading, tightly coupled with fluid supply and exchange as well as interaction with body systems (intervertebral disk, cartilageous end plate, larynx, etc.). Proper test conditions and data processing can assist the development of better scaffolds for cartilage repair.

## Author Contributions

The manuscript has been written and composed by MG and AZ, with the assistance of all other authors. Materials preparation and description were done by A-MH and KL under guidance of MK. Medical relevance has been analyzed by IK and VM, and data from parallel animal studies were obtained and processed by VM. Advanced data processing was performed by YB based on experiments made by AZ. All authors have reviewed the manuscript.

### Conflict of Interest Statement

MG is a shareholder of company Seqvera Ltd.

The remaining authors declare that the research was conducted in the absence of any commercial or financial relationships that could be construed as a potential conflict of interest.

## References

[B1] Agrawal C. M. Parr J. E. Lin S. T. (2000). Synthetic Biodegradable Polymers for Implants. West Conshohocken, PA: ASTM STP1936.

[B2] Ahsan T. Sah R. L. (1999). Biomechanics of integrative cartilage repair. Osteoarthr. Cartil. 7, 29–40. 10.1053/joca.1998.0160 10367013

[B3] Armstrong C. G. Mow V. C. (1982). Variations in the intrinsic mechanical properties of human articular cartilage with age, degeneration, and water content. J. Bone Joint Surg. 64, 88–94. 10.2106/00004623-198264010-00013 7054208

[B4] Benders K. E. van Weeren P. R. Badylak S. F. Saris D. B. Dhert W. J. Malda J. (2012). Extracellular matrix scaffolds for cartilage and bone regeneration. Trends Biotechnol. 31, 169–176. 10.1016/j.tibtech.2012.12.004 23298610

[B5] Bennethum L. S. Cushman J. H. (1996). Multiscale, hybrid mixture theory for swelling systems–II: constitutive theory. Int. J. Eng. Sci. 34, 147–169.

[B6] Bennethum L. S. Weinstein T. (2004). Three pressures in porous media. Transp. Porous Media 54, 1–34. 10.1023/A:1025701922798

[B7] Bilotsky Y. Gasik M. (2015). Modelling of poro-visco-elastic biological systems. J. Phys. Conf. Series 633:021234 10.1088/1742-6596/633/1/012134

[B8] Burdick J. A. Mauck R. L. (2011). Biomaterials for Tissue Engineering Applications: A Review of the Past and Future Trends. New York, NY: Springer, 564 10.1007/978-3-7091-0385-2

[B9] Chung C. Burdick J. A. (2008). Engineering cartilage tissue. Adv. Drug Deliv. Rev. 60, 243–262. 10.1016/j.addr.2007.08.027 17976858 PMC2230638

[B10] Directive 2010/63/EC for Alternative Methods (2015). Directive 2010/63/EC for Alternative Methods “3R” = Replacement, Reduction, Refinement, Complemented With ‘Stop Vivisection' The European Citizens'. Initiative submitted to the European Commission (C(2015)3773 final).

[B11] Fam H. Bryant J. T. Kontopoulou M. (2007). Rheological properties of synovial fluids. Biorheology 44, 59–74. 17538199

[B12] Frost H. M. (2004). A 2003 update of bone physiology and Wolff's law for clinicians. Angle Orthodont. 74, 3–15. 10.1043/0003-3219(2004)074<0003:AUOBPA>2.0.CO;2 15038485

[B13] Gasik M. (2014). New BEST – biomaterials enhanced simulation test. J. Tissue Sci. Eng. 5:66 10.4172/2157-7552.S1.014

[B14] Gasik M. (2017a). In vitro Test Method for Implant Materials. Patent US 9683267B2.

[B15] Gasik M. (2017b). Understanding biomaterial-tissue interface quality: combined *in vitro* evaluation. Sci. Techn. Adv. Mater. 18, 550–562. 10.1080/14686996.2017.1348872 28970865 PMC5613488

[B16] Gasik M. Bilotsky Y. (2018). High-output screening and biomechanical optimization of biomaterials for orthopaedic applications. Orthopaed. Proc. 100B:S44S3.

[B17] Gasik M. Hiropoulos I. Zühlke A. Muhonen V. Haaparanta A.-M. Laine K. (2017). Biomechanical comparison for commercial and novel scaffolds for articular cartilage repair. Orthopaed. Proc. 99B(Suppl. 1), 79.

[B18] Gerisch A. Chaplain M. A. J. (2006). Robust numerical methods for taxis-diffusion-reaction systems: applications to biomedical problems. Mathem. Comput. Model. 43, 49–75. 10.1016/j.mcm.2004.05.016

[B19] Gomes M. E. Reis R. L. (2004). Biodegradable polymers and composites in biomedical applications: from catgut to tissue engineering. Intern. Mater. Rev. 49, 261–273. 10.1179/095066004225021918

[B20] Goode R. L. Shinn J. B. (1977). Long-term stenting in the treatment of subglottic stenosis. Ann. Otol. Rhinol. Laryngol. 86(6 Pt 1), 795–8. 413464 10.1177/000348947708600613

[B21] Gunawardena J. (1996). An Introduction to Idempotency. Bristol, UK: HP Laboratories Bristol, Publication HPL-BRIMS-96-24 p. 50.

[B22] Haaparanta A. M. Järvinen E. Cengiz I. F. Ellä V. Kokkonen H. T. Kiviranta I.. (2014). Preparation and characterization of collagen/PLA, chitosan/PLA, and collagen/chitosan/PLA hybrid scaffolds for cartilage tissue engineering. J. Mater. Sci. Mater. Med. 25, 1129–1136. 10.1007/s10856-013-5129-5 24375147

[B23] Hayes W. (1972). Some viscoelastic properties of human articular cartilage. Acta Orthop. Belg. 38, 23–31. 4656162

[B24] Hayes W. C. Bodine A. J. (1978). Flow-independent viscoelastic properties of articular cartilage matrix. J. Biomech. 11, 407–419. 10.1016/0021-9290(78)90075-1 213441

[B25] Hayes W. C. Mockros L. F. (1971). Viscoelastic properties of human articular cartilage. J. Appl. Physiol. 31, 562–568. 10.1152/jappl.1971.31.4.562 5111002

[B26] Hosseini S. M. Wilson W. Ito K. van Donkelaar C. C. (2014). How preconditioning affects the measurement of poro-viscoelastic mechanical properties in biological tissues. Biomech. Model. Mechanobiol. 13, 503–513. 10.1007/s10237-013-0511-2 23864393

[B27] Hubbell J. A. (1995). Biomaterials in tissue engineering. Nat. Biotechnol. 13, 565–576. 10.1038/nbt0695-565 9634795

[B28] Kempson G. E. Freeman M. A. Swanson S. A. (1971). The determination of a creep modulus for articular cartilage from indentation tests of the human femoral head. J. Biomech. 4, 239–250. 10.1016/0021-9290(71)90030-3 5122817

[B29] King R. G. (1966). A rheological measurement of three synovial fluids. Rheol Acta 5, 41–44. 10.1007/BF01973577

[B30] Kobayashi Y. Okamoto A. Nishinari K. (1994). Viscoelasticity of hyaluronic acid with different molecular weights. Biorheology 31, 235–244. 10.3233/BIR-1994-31302 8729484

[B31] Korhonen R. K. Julkunen P. Rieppo J. Lappalainen R. Konttinen Y. T. Jurvelin J. S. (2006). Collagen network of articular cartilage modulates fluid flow and mechanical stresses in chondrocyte. Biomech. Model. Mechanobiol. 5, 150–159. 10.1007/s10237-006-0021-6 16506019

[B32] Korhonen R. K. Laasanen M. S. Töyräs J. Rieppo J. Hirvonen J. Helminen H. J.. (2002). Comparison of the equilibrium response of articular cartilage in unconfined compression, confined compression and indentation. J. Biomech. 35, 903–909. 10.1016/S0021-9290(02)00052-0 12052392

[B33] Lai W. M. Hou J. S. Mow V. C. (1991). A triphasic theory for the swelling and deformation behaviors of articular cartilage. J. Biomech. Eng. 113, 245–258. 10.1115/1.2894880 1921350

[B34] Lai W. M. Mow V. C. Roth V. (1981). Effects of nonlinear strain-dependent permeability and rate of compression on the stress behavior of articular cartilage. J. Biomech. Eng. 103, 61–66. 10.1115/1.3138261 7278183

[B35] Länsman S. Pääkkö P. Ryhänen J. Kellomäki M. Waris E. Törmälä P.. (2006). Poly-L/D-lactide (PLDLA) 96/4 fibrous implants: histological evaluation in the subcutis of experimental design. J. Craniofac. Surg. 17, 1121–1128. 10.1097/01.scs.0000231627.33382.85 17119416

[B36] Litvinov G. L. Maslov V. P. Shpiz G. B. H. (2001). Idempotent functional analysis: an algebraic approac. Mathem. Notes 69, 696–729. 10.1023/A:1010266012029

[B37] Lubarda V. A. Chen M. C. (2008). On the elastic moduli and compliances of transversely isotropic and orthotropic materials. J. Mech. Mater. Struct. 3, 153–171. 10.2140/jomms.2008.3.153

[B38] Lujan T. J. Wirtz K. M. Bahney C. S. Madey S. M. Johnstone B. Bottlang M. (2011). A novel bioreactor for the dynamic stimulation and mechanical evaluation of multiple tissue-engineered constructs. Tissue Eng. C 17, 367–374. 10.1089/ten.tec.2010.0381 20950252 PMC3045075

[B39] Mak A. F. Lai W. M. Mow V. C. (1987). Biphasic indentation of articular cartilage—I. Theoretical analysis. J. Biomech. 20, 703–714. 10.1016/0021-9290(87)90036-4 3654668

[B40] Mäkelä J. T. A. Korhonen R. K. (2016). Highly non-linear stress-relaxation response of articular cartilage in indentation: importance of collagen nonlinearity. J. Biomech. 49, 1734–1741. 10.1016/j.jbiomech.2016.04.00 27130474

[B41] Maslov V. P. (1970). The characteristics of pseudo-differential operators and difference schemes. Actes Congrès. Intern. Math. 2, 755–769.

[B42] Milan J. L. Planell J. A. Lacroix D. (2010). Simulation of bone tissue formation within a porous scaffold under dynamic compression. Biomech. Model. Mechanobiol. 9, 583–596. 10.1007/s10237-010-0199-5 20204446

[B43] Mollon B. Kandel R. Chahal J. Theodoropoulos J. (2013). The clinical status of cartilage tissue regeneration in humans. Osteoarthr. Cartil. 21, 1824–1833. 10.1016/j.joca.2013.08.024 24018339

[B44] Mow V. C. Kuei S. C. Lai W. M. Armstrong C. G. (1980). Biphasic creep and stress relaxation of articular cartilage in compression: theory and experiments. J. Biomech. Eng. 102, 73–84. 10.1115/1.3138202 7382457

[B45] Muhonen V. Salonius E. Haaparanta A. M. Järvinen E. Paatela T. Meller A.. (2016). Articular cartilage repair with recombinant human type II collagen/polylactide scaffold in a preliminary porcine study. J. Orthopaed. Res. 34, 745–753. 10.1002/jor.23099 26573959

[B46] Norris A. (2008). Eulerian conjugate stress and strain. J. Mech. Mater. Struct. 3, 243–260. 10.2140/jomms.2008.3.243

[B47] Panadero J. A. Lanceros-Mendez S. Gomez Ribelles J. L. (2016). Differentiation of mesenchymal stem cells for cartilage tissue engineering: individual and synergetic effects of three-dimensional environment and mechanical loading. Acta Biomater. 33, 1–12. 10.1016/j.actbio.2016.01.037 26826532

[B48] Pioletti D. P. Rakotomanana L. R. (2000). Non-linear viscoelastic laws for soft biological tissues. Eur. J. Mech. A Solids 19, 749–759. 10.1016/S0997-7538(00)00202-3

[B49] Pulliainen O. Vasara A. I. Hyttinen M. M. Tiitu V. Valonen P. Kellomäki M.. (2007). Poly-L-D-lactic acid scaffold in the repair of porcine knee cartilage lesions. Tissue Eng. 13, 1347–1355. 10.1089/ten.2006.0347 17518746

[B50] Sittichokechaiwut A. Edwards J. H. Scutt A. M. Reilly G. C. (2010). Short bouts of mechanical loading are as effective as dexamethasone at inducing matrix production by human bone marrow mesenchymal stem cells. Eur. Cells Mater. 20, 45–57. 10.22203/eCM.v020a05 20648425

[B51] US FDA (2010). Guidance for the Use of Bayesian Statistics in Medical Device Clinical Trials. Guidance for industry and FDA staff. US FDA Docket No.2006D-0191, 50.

[B52] Volfson D. Cookson S. Hasty J. Tsimring L. S. (2008). Biomechanical ordering of dense cell populations. PNAS 105, 15346–15351. 10.1073/pnas.0706805105 18832176 PMC2563119

[B53] von Recum A. (1998). Handbook of Biomaterials Evaluation, ed. (New York, NY: Taylor-Francis), 916.

[B54] Wilson W. Huyghe J. M. van Donkelaar C. C. (2006). A composition-based cartilage model for the assessment of compositional changes during cartilage damage and adaptation. Osteoarthr. Cartil. 14, 554–560. 10.1016/j.joca.2005.12.006 16476555

[B55] Wilson W. van Donkelaar C. C. Huyghe J. M. (2005). A comparison between mechano-electrochemical and biphasic swelling theories for soft hydrated tissues. J. Biomech. Eng. Trans. ASME 127, 158–165. 10.1115/1.1835361 15868798

[B56] Wong J. Y. Bronzino J. D. (2007). Biomaterials (Boca Raton, FL: CRC Press; Taylor & Francis), 296 10.1201/9780849378898

[B57] Xiao H. (1995). Invariant characteristic representations for classical and micropolar anisotropic elasticity tensors. J. Elasticity 40, 239–265.

